# Chaplain Care in the Intensive Care Unit at the End of Life: A Qualitative Analysis

**DOI:** 10.1089/pmr.2021.0012

**Published:** 2021-10-18

**Authors:** Ian McCurry, Pauline Jennett, Jimin Oh, Betty White, Horace M. DeLisser

**Affiliations:** ^1^ Academic Programs Office and the Pulmonary, Allergy and Critical Care Division, Department of Medicine, Perelman School of Medicine, University of Pennsylvania, Philadelphia, Pennsylvania, USA.; ^2^ Department of Pastoral Care and Education, University of Pennsylvania Health System, Philadelphia, Pennsylvania, USA.; ^3^ Graduate School of Education, University of Pennsylvania, Philadelphia, Pennsylvania, USA.

**Keywords:** chaplains, end-of-life decision making, intensive care unit, pastoral care, spirituality

## Abstract

***Background:*** The provision of spiritual care is a key component of high-quality patient-centered care, particularly in the intensive care unit (ICU). However, the integration of spiritual care into the care of patients in the ICU is variable, especially at the end of life, which may be due in part to poor or incomplete provider knowledge of the work of chaplains.

***Objective:*** To characterize the care and services provided by chaplains to patients in an ICU at the end of life and/or their families.

***Design:*** A retrospective chart review was performed to identify all patients admitted over a three-month period to an ICU who had visits with a chaplain and an ICU course that ended in death, discharge to a palliative care facility or discharge to hospice.

***Subjects/setting:*** Twenty-five chaplains at a U.S. medical center.

***Measurements:*** Qualitative analysis was performed using directed content analysis on the notes written by the chaplains.

***Results:*** Qualitative analyses of the chaplain notes revealed four broad themes regarding the activities of chaplains in the ICU with respect to patients and families. These were that chaplains provide comfort to patients and family facing the end of life, provide prayers with a variety of purposes, assist in supporting family members through complex medical decision making, and provide connections to appropriate resources.

***Conclusions:*** Chaplains contribute to the care of patients in the ICU through a wide range of activities that demonstrate the unique intermediary and collaborative role chaplains can play within the health care team at the end of life in the ICU.

## Introduction

The provision of spiritual care is recognized as a key component of high-quality patient-centered care.^1,2^ Although some providers, notably nurses,^3^ may be skilled generalists in the provision of spiritual care, chaplains are specialists in this role,^4–6^ providing spiritual presence, supporting and nurturing relationships across diverse populations and experiences, and aiding in creating meaning in the illness experience.^7,8^ Provision of spiritual care by chaplains has been linked to improved satisfaction and decreased expenditures at the end of life.^8–12^ Furthermore, there is evidence that patients who receive care in hospitals with integrated spiritual care services have significantly lower rates of hospital death and higher rates of hospice enrollment.^13^

In the setting of the intensive care unit (ICU), the need for spiritual care is pressing given the burdens of end-of-life decision making and the relational, spiritual, and existential challenges faced by patients and their families.^14,15^ Despite the recognized need, the integration of spiritual care into the care of patients in the ICU is variable.^16–19^ The reasons for this are likely several, but may reflect poor or incomplete provider knowledge of the work of chaplains.^20–22^ However, several recent published reports have begun to provide a more robust understanding of the activities of chaplains in the ICU that may help to address these gaps in provider knowledge.^19,23–25^ Our article adds to that literature by describing the work of ICU chaplains as seen through the contemporaneous notes in the medical records of their visits or interactions with patients and/or families.

## Materials and Methods

### Study design

The purpose of the study was to characterize the care and services provided by chaplains to patients in an ICU at the end of life and/or their families. It involved a retrospective chart review of the electronic medical record (EMR) of all patients admitted between February and April of 2017 to the Medical ICU (MICU) of the Hospital of the University of Pennsylvania (HUP), an 800-bed regional tertiary academic medical center. The HUP MICU is a 24-bed closed unit, caring for adult noncardiac nonsurgical critically ill patients. The pastoral care program at HUP consists of a mix of chaplain trainees of varying levels of experience and workload as well as staff chaplains who work regularly within the unit. The full-time chaplains conduct rounds on all patients each morning, and night-time chaplains perform focused rounds based on recommendations from nursing leadership. Chaplains are also available through pager 24 hours daily for acute needs. Night and weekend coverage are provided by a rotation of adjunct chaplains who work one to two shifts per month.

Inclusion criteria for the study were met if the patient received care in the MICU, had visits with a chaplain documented in the EMR, and the ICU stay progressed to an “end-of-life” outcome. These outcomes included death in the ICU, discharge to a palliative care facility, or discharge to hospice. Patient charts were reviewed, and demographics and clinical course extracted. In addition, chaplain demographics were also obtained, including gender, race, level of education, religion, and frequency of chaplain practice. The study was reviewed and approved by the Institutional Review Board of the University of Pennsylvania.

### Data analysis

Qualitative analysis was performed using directed content analysis on the notes written by chaplains contained within the EMR. Preliminary codes were initially established by one of the authors (I.M.). These analytic categories were subsequently modified as the analysis continued based on the data.^26^ Secondary coding was performed with all members of the research team to ensure accuracy of codes derived.^27^ An audit trail was maintained throughout formation of the code list to strengthen the scientific rigor of the analysis.^28^ After completion and review of the coding, individual codes were grouped together to form specific categories, which were compiled into broad themes.^28^ After ∼65 charts were reviewed no new codes were established, indicating saturation of the data.^29^ Descriptive demographic statistics were performed using IBM SPSS Statistics 24 for windows.

## Results

### Chaplain and patient demographics

There were 25 chaplains whose notes were analyzed in this study, derived from 121 unique patients, with 95 patients having more than one chaplain note that was included in the study. The chaplain and patient demographic information are presented, respectively, in Tables 1 and 2.

**Table 1.** Demographic Characteristics of Chaplains (*N* = 25)

Characteristic	Chaplains, n (%)
Gender	
Male	10 (40)
Female	15 (60)
Age	
Average	52.8
Race	
Black	12 (48)
White	11 (44)
Asian	2 (8)
Education	
Bachelor's degree	4 (16)
Master of theology	1 (4)
Master of divinity	16 (64)
Rabbinical school	1 (4)
Doctorate	3 (12)
Religion	
Baptist	9 (36)
Buddhist	2 (8)
Evangelical	1 (4)
Jewish	2 (8)
Lutheran	1 (4)
Methodist	1 (4)
Nondenominational	4 (16)
Pentecostal	2 (8)
Seventh-day Adventist	1 (4)
United Church of Christ	2 (8)
Workload	
Adjunct	18 (72)
Part time	4 (16)
Full time	3 (12)

**Table 2.** Characteristics of Patients (*N* = 121)

Characteristic	Patients, n (%)
Gender	
Male	55 (45)
Female	66 (55)
Race	
Black	29 (24)
White	76 (63)
Asian	3 (2)
Other	13 (11)
Religion	
Baptist	12 (10)
Church of God	3 (2)
Jewish	5 (4)
Lutheran	3 (2)
Methodist	2 (2)
Muslim	2 (2)
Nondenominational	3 (2)
Other	10 (8)
Protestant	4 (3)
Roman Catholic	37 (31)
Unknown	40 (33)
Outcome	
Death	86 (71)
Hospice	18 (15)
Other care facility	17 (14)

### Qualitative data on ICU chaplain activities

Although the notes of 25 different chaplains were analyzed for this study, 5 full-time chaplains provided the majority of longitudinal care to patients in the ICU and wrote the majority of the notes (76%). With respect of the nature of the documentation, some of the chaplains provided brief action-based reports, whereas others wrote more extensive narrative descriptions of the care provided. The longer notes tended to be written by more experienced full-time chaplains, whereas part-time and less experienced chaplains tended to write shorter more objective notes. Information on who specifically requested a chaplain visit was not routinely or consistently noted.

Qualitative data analysis of the chaplain notes in the medical records revealed four broad themes regarding the activities of chaplains in the MICU with respect to patients and families. These were that chaplains provide comfort to patients and family facing the end of life, provide prayers with a variety of purposes, assist in supporting family members through complex medical decision making, and provide connections to appropriate resources. These are discussed further in more detail hereunder.

### Theme 1: Chaplains provide comfort to patients and family facing the end of life

“I visited with the patient's family after a rapid response was called. Her daughter was outside the room during the episode and was visibly shaken/tearful. She shared with me her father's journey and her fears from an uncertain diagnosis. I offered listening presence, questions for reflection, and an emotionally supportive presence.”

Chaplains frequently noted the provision of “comfort” in their documentation. This came in various forms depending on the nature of the encounter. In situations where patients were still responsive, chaplains reported providing spiritual comfort through reflective listening to patients' spiritual distress to create meaning in the course of their illness. In these conversations, the chaplains assisted in preparing for changes in functional status and processing concerns about pain at the end of life and burdens placed on caregivers.

In some encounters, chaplains were called to visit an unconscious patient to provide spiritual care to the patient's family and the health care team. This often included spiritual presence, comforting words, and occasionally therapeutic touch and embrace. Chaplains also provided comforting food and drink to family members in collaboration with the guest services at the hospital.

When family and staff were not present, chaplains noted they provided silent prayer and mindful presence with an otherwise alone patient. Several notes mentioned that other members of the team thanked them for this service.

Finally, chaplains often comforted family members through the creation of “safe spaces.” Emotional safe spaces were created through chaplain-based mediation of conflicts between the patient and family and assistance with understanding family dynamics, whereas the reservation of family waiting rooms established physical safe spaces for families to be together and express emotions privately.

### Theme 2: Chaplains provide prayers with a variety of purposes

“I was called to the gathering, so I assumed there had been a significant change. Instead, he was much more interactive today. Family was close by and expressed hope. I prayed a prayer for encouragement and healing then excused myself as I felt they were sharing something very intimate.”

The leading of prayers was frequently mentioned in the chaplain notes, with five “types” of prayer being offered.

#### Prayers for strength

Chaplains noted praying for the strength of patients in the face of their illness. They also noted several families asked them to pray for the strength to face change, whether it be death or transition into the dying process.

#### Prayer as a reflective listening tool

Chaplains noted summarizing family priorities and discussions in the form of prayer. After lengthy discussions with family members covering the patient's past life, course of illness, tenuous family dynamics, and/or priorities in treatment, chaplains reported summarizing these themes into a cohesive prayer with the family. Allowing the family to contribute to these prayers further served to ensure that family preferences and priorities had been accurately captured by the chaplain.

#### Prayer for healing

Spiritual care providers frequently prayed for healing from physical suffering and emotional burdens as well as offered prayers for providers to have the skill and competence in providing healing care. Review of the notes revealed that prayers for physical healing were not so much requests for divine intervention but efforts to align with patient or family hope and wishes for a different outcome in the face of bleak medical realities.

#### Prayers to express grief

Family members requested prayers of bereavement. These prayers were often requested in conjunction with those for comfort while patients were facing the end of life or had already passed.

#### Prayers to express gratitude

Chaplains documented prayers of thanksgiving for lives well lived and peaceful deaths. They also noted prayers of gratitude for patients' lifelong contributions to their families. Others documented prayers of expressed appreciation for spiritual well-being, comfort, and wisdom.

### Theme 3: Chaplains assist in supporting family members through complex medical decision making

“I asked what [the patient] was hoping for from this and she indicated she wanted to return home and be with her family. She was hoping to continue to wean her ventilator but felt hopeless that she might be moving too slowly towards the goal.”

Chaplains were frequently called upon to support families making end-of-life decisions in one of three ways. First, when decisions involved the use of invasive therapies such as ventilators, renal replacement, and feeding tubes, the chaplains facilitated the patient and/or families understanding of these technologies and how they did or did not align with patient/family goals. Second, they advocated for families in the decision-making process. This included speaking with the medical team on behalf of families to foster better communication and initiating delays in transitions of care to allow for family members to arrive or the provision of religious rituals such as last rites. Finally, the chaplains facilitated the creation of meaning within the context of the illness decision-making process. This was done through discussions of past priorities and desires of patients. Chaplains frequently provided the vocabulary and relational tools to clarify the spiritual and ethical tension for the patient/family. They also provided validation to the patient/family that enabled them to move forward with their decisions.

### Theme 4: Chaplains provide connections to appropriate resources

“Family has decided to transition to comfort care, requested that a catholic priest deliver the sacrament of the sick. Referral placed to on-call priest.”

Chaplains documented connecting patients and family members to various resources. One chaplain referred multiple patients with intractable pain to the hospital's Reiki provider. Chaplains also provided guidance in contacting funeral homes to arrange for culturally appropriate services. When patients expressed psychospiritual stress from housing and food insecurity, chaplains often reached out to hospital social workers to assist these patients.

When patients expressed anxiety surrounding their illness, chaplains provided patient education about relaxation techniques, including breathing exercises and mindfulness. This was accomplished through verbal teaching and referral to other written and video sources. When patients were found to be acutely anxious, the medical team was alerted to provide mental health support.

Patients also frequently requested rituals pertinent to a specific religious belief. In some instances, these might be provided by the chaplain, such as provision of the sacrament of the sick for Catholic patients. When this was not possible, or not desired by the patient, the chaplains facilitated connections through an established referral network or to the spiritual community of the patient or family.

## Discussion

Our data indicate that chaplains contribute to the care of patients in the ICU through a wide range of activities that include providing comfort to patients and family facing the end of life, providing prayers with a variety of purposes, assisting in supporting family members through complex medical decision making, and providing connections to appropriate resources. These activities speak to the unique intermediary and collaborative role chaplains can play within the health care team at the end of life in the ICU.

Several studies in recent years have provided descriptions of the types of activities and services provided by chaplains in the ICU.^8,19,23,24^ In a study of spiritual care providers surveyed after the death of patients at one institution, discussions with families about spiritual and religious needs, family member feelings, patient values, and reminiscences of the patient were common (>80% of the interactions).^19^ More specific details regarding the nature of these conversations were not provided. In their retrospective review, Lee et al. focused on the nature and quality of ICU chaplain documentation, noting that the chaplains they studied tended to use “coded language” that failed to capture the details of what they did or the fullness of patient/family stories.^23^ The study by Labuschagne et al., a retrospective observation study of spiritual care at three academic medical centers and one faith-based community hospital, provided detailed quantitative data on the types of spiritual care activities during chaplain visits.^24^ In this cohort of chaplains, provision of emotional/spiritual support and ritual/prayer/religious resources were common (>50% of visits). The data from our study complement and extend the findings of these previous reports, by providing a more granular qualitative description of the work of chaplains in the ICU.

In describing the activities of chaplains in the ICU, this article also raises issues more broadly about chaplain identity. Norwood^30^ discussed the concept of an “ambivalent chaplain” in which the chaplain alternates between embracing their connection to spiritual or religious paradigms when working with families and engaging medical frameworks when speaking to clinicians. This ability to move seamlessly between the spiritual and the clinical makes the seasoned chaplain well positioned to work with families when the medical team feels ill-equipped to address the relational, spiritual, or existential distress of families.

Our data demonstrate that the services provided by chaplains in the ICU are multiple, some of which are or could be delivered by other providers. This raises the important question of what are the potentially unique contributions that chaplains can make to ICU care? In response, we posit that the chaplain is able to understand both the pertinent clinical issues as well as the spiritual distress they cause families in ways that nurses, physicians, and advance practice providers are unable to do. Although ICU providers can affirm and support patient/family spirituality, the lack of time and training limit their ability to effectively bridge the clinical and the spiritual domains of care. Undistracted by the demands of providing clinical care required of ICU providers, chaplains are able to focus their patient/family care time on identifying spiritual distress, burdens and issues, and then addressing them through comfort, prayer, decision-making support, and connections to resources.^31,32^ Importantly, our analysis confirms the chaplain's role of being “dedicated listener,” as discussed by Handzo.^31^ The experience of being fully listened to serves as an important therapeutic intervention before any action such as referral to resources or offering of prayer.^31,33^

Several limitations are noted. First, this study only analyzed the documentation of chaplains from one MICU. Furthermore, the majority of the notes analyzed were from a cohort of five chaplains who provided the majority of longitudinal care to patients in the MICU. Therefore, this study may not be representative of the work done by chaplains at the end of life in other ICU settings and/or at other institutions. Second, there were variations in documentation styles among the chaplains, ranging from brief action-based reports to narrative descriptions. Consequently, these chaplain notes, although contemporaneous with the services provided, may not fully capture the activities of the MICU chaplain. To address this issue, future studies should be prospective using a templated note to standardize the documentation or employ ethnographic approaches. Finally, this study did not explore the impact of chaplain activities and how they are perceived by patients or their families, nor the care that chaplains provide to ICU staff, and issues that should be addressed in future investigations of the work of ICU chaplains.

## Conclusion

Our data highlight the particularly valuable role that ICU chaplains can play in contributing to interdisciplinary care to critically patients and their families and those who care for them. Furthermore, reports such as ours may be helpful in alerting ICU providers to the work of chaplains in the ICU and their contributions to patient care, and in turn prompt both individual and institutional efforts at integrating chaplains more intentionally into the care of critically ill patients. Providers often have a limited or distorted understanding of the role of chaplains in clinical care,^20–22,34^ although there are efforts occurring in medical education that seek to inform trainees about the work of chaplains.^35–37^ As the scope and nature of ICU chaplaincy evolves to include more explicit notions of advocacy and patient navigation,^38,39^ it becomes even more imperative that clinicians are better informed of the work of chaplains to enable them to collaborate more effectively with chaplains.

## Abbreviations Used

EMR: electronic medical record
HUP: Hospital of the University of Pennsylvania
ICU: intensive care unit
MICU: Medical ICU
